# Viscoelastic Damping in alternate reciprocating contacts

**DOI:** 10.1038/s41598-017-08507-8

**Published:** 2017-08-21

**Authors:** Carmine Putignano, Giuseppe Carbone

**Affiliations:** Department of Mechanics, Mathematics and Management, Politecnico di Bari, Bari, Italy

## Abstract

Reciprocating motion between viscoelastic solids occurs in a number of systems and, in particular, in all the dampers which exploits, as a physical principle, the viscoelastic dissipation. So far, any attempt to predict the behavour of this field of dampers relies on approximate methodologies and, often, on a steady-state approach, with a consequent poor understanding of the phenomenon. Here, we develop a methodology capable of simulating the actual mechanics of the problem and, in particular, we shed light on how the presence of not fully relaxed viscoelastic regions, during the punch motion, determine the viscoelastic dissipation. The latter is shown to be dependent ultimately on two dimensionless parameters, i.e. the maximum speed in the cycle and the frequency. Finally, the importance of considering a rough interface is enlightened.

## Introduction

The assessment of energy dissipation and losses, in all their different forms, including, for example, friction, phase transitions, material hysteresis, is crucial in current applied science and engineering research. From a mechanical point of view, modern design craves, in fact, procedures and tools to understand how to minimize friction and, consequently, to increase the efficiency of components and systems. However, there are still many and prominent fields, where dissipating energy is, on the contrary, sought and represents a strict issue in the design specifications. Indeed, curbing any source of mechanical noise and, more specifically, damping vibrations are essential in a countless number of applications and have been pursued by means of a variety of configurations, including e.g. viscous^1^, sliding^2^, magnetorheological^3^ dampers. Indeed, to this extent, particularly interesting is the case of systems that exploit the viscoelastic hysteresis, i.e. the energy dissipation occurring when a solid with a viscoelastic rheology undergoes a periodic deformation. Given the physical principle which viscoelastic dampers relies on, i.e. the mechanical deformation of contacting viscoelastic bodies, these devices, usually made of rubber-based composites, have a broad diffusion in a range of scales and application fields: possible examples include dampers in microelectronics^4^, in automotive technology for noise reduction^5^, or in seismic engineering to reduce the consequences of earthquakes^6, 7^. The main drawback of these systems, however, is the lack of reliable theoretical models. Currently, the design in the field mostly relies on practical and empirical guidelines, and no tool is available to get quantitative predictions. This is mainly due to the strongly time-dependent constitutive stress-strain relations that govern the problem and have to be managed when dealing with viscoelastic dissipation, both in terms of friction and damping, in contact mechanics.

Given the practical interest and, at the same time, the related intricacy of the problem, many research efforts have been dedicated to the viscoelastic dissipation: these include analytical^8–10^, numerical^11, 12^ and experimental^13–15^ analyses. In particular, in the last two decades, a large part of the research activity has been dedicated to investigate unidirectional steady-state sliding between two mating viscoelastic bodies^9, 12, 16^. As a matter of fact, we are able to focus on steady-state sliding in a range of conditions: these include, for example, the presence of rough interfaces^17, 18^, of inclusions^19^ or of border effects^20^. However, steady-state assumptions cannot be considered universally valid given the countless number of engineering applications, including, in particular, any damping system, where the motion direction is periodically inverted. The reciprocating contact mechanics of viscoelastic materials is a field that has started to be studied only very recently^21^ and needs to be further investigated.

The aim of this paper is to shed additional light on the reciprocating motion and, in particular, on how the damping is influenced by the main parameters marking the reciprocating contact mechanics, i.e. the amplitude, the frequency and the load. In order to carry out the analysis, we move from the Boundary Element methodology (BEM), originally developed in ref. 21, and enhance such an approach by means of a FFT-based method enabling a significant speed up in the BEM Green’s function calculation. Furthermore, in this study, we underline how, in the case of an alternative motion, an estimation based on steady-state assumptions leads to errors that can be really large. This is even more significant in the presence of a rough interface, which tends to increase the dissipated energy. The paper is outlined as follows. Section II describes the mathematical formulation implemented in the Boundary Element method. Section III include the results in terms of deformation - the dissipated power is indeed proportional to deformed volume- and in terms of the tangential force in a range of different conditions of frequency and maximum speed. Furthermore, the role of the roughness is discussed. Final remarks close the paper and comment on the significance of the reciprocating regime to assess the dissipated power.

## Formulation

By recalling the translational invariance and the elastic-viscoelastic correspondence principle^22^, the general linear-viscoelastic contact problem between a rigid indenter and a viscoelastic slab can be formulated by means of the following integral equations relating interfacial stresses and strains:

1 $$u({\bf{x}},t)={\int }_{-\infty }^{t}d\tau \int {d}^{2}{x}^{^{\prime} }{\mathscr{J}}(t-\tau ){\mathscr{G}}({\bf{x}}-{\bf{x}}{\boldsymbol{^{\prime} }})\dot{\sigma }({\bf{x}}{\boldsymbol{^{\prime} }},\tau ),$$

where **x** is the in-plane position vector, *t* is the time, *u*(**x**, *t*) and *σ*(**x**, *t*) are respectively the normal surface displacement of the viscoelastic solid and the normal interfacial stress, ${\mathscr{G}}({\bf{x}})$ and ${\mathscr{J}}(t)$ are the Green’s function and the creep function, and the symbol ‘·’ refers to the Lagrangian time derivative. The latter quantity is the creep function which satisfies causality, i.e. ${\mathscr{J}}(t < 0)=0$, and, for a generic viscoelastic material^22^, can be written as:

2 $${\mathscr{J}}(t)= {\mathcal H} (t)[\frac{1}{{E}_{0}}-{\int }_{0}^{+\infty }d\tau {\mathscr{C}}(\tau )\exp (-t/\tau )]$$

where ${\mathcal H} (t)$ is the Heaviside step function, the real quantity *E*_0_ is the low-frequency elastic modulus, ${\mathscr{C}}(\tau )$ is a strictly positive function usually defined as the creep (or retardation) spectrum^22, 23^, and *τ* is the relaxation time, continuously distributed on the real axis.

Now, solving directly Eq. (1) requires to discretize both the time and the space domains and this may be unfeasible in a number of cases, and, remarkably, when roughness has to be accounted for. Indeed, given a rough spectrum, covering typically several orders of magnitude, the number of boundary elements required would be too large for the computational resources currently available.

However, since we are going to study a reciprocating motion, given the periodicity of the system, we can try to simplify Eq. (1). To this end, let us start assuming that the interfacial normal stress distribution *σ*(**x**, *t*) is equal to $\sigma ({\bf{x}},t)=\sigma [{\bf{x}}-{\xi }_{0}\,\sin (\omega t)]$: this means that the shape of interfacial distribution is time-indipendent and moves on the viscoelastic half-space with a sinusoidal law of amplitude $|{\xi }_{0}|$ and angular frequency *ω*. Now, because of the linearity and the translational invariance characterizing the problem, we can carry out the replacement ${\bf{x}}\to {\bf{x}}+{\xi }_{0}\,\sin (\omega t)$, and therefore Eq. (1) can be rewritten as:

3 $$u({\bf{x}},t)=\int {d}^{2}x^{\prime} G({\bf{x}}-{\bf{x}}{\boldsymbol{^{\prime} }},t)\sigma ({\bf{x}}{\boldsymbol{^{\prime} }})\mathrm{.}$$

A significant advantage is embedded in Eq. (3): there exists only a parametric dependence on the time and, therefore, only the space domain has to be discretized to solve completely the problem.

Now, *G*(**x**, *t*) has to be determined. To this end, as shown in refs 12 and 21, it is possible to recall the general relation between stress and displacement distributions:

4 $$u({\bf{x}},t)={\mathscr{J}}(0)\int {d}^{2}x^{\prime} {\mathscr{G}}({\bf{x}}-{\bf{x}}{\boldsymbol{^{\prime} }})\sigma ({\bf{x}}{\boldsymbol{^{\prime} }},t)+{\int }_{-\infty }^{t}d\tau \dot{{\mathscr{J}}}(t-\tau )\int {d}^{2}x^{\prime} {\mathscr{G}}({\bf{x}}-{\bf{x}}{\boldsymbol{^{\prime} }})\sigma ({\bf{x}}{\boldsymbol{^{\prime} }},\tau ),$$

Furthermore, given the constant shape assumption previously stated, we can replace *σ*(**x**, *t*) with $\sigma ({\bf{x}},t)=\delta [{\bf{x}}-{\xi }_{0}\,\sin (\omega t)]$ in Eq. (3) and, then, obtain:

5 $$G({\bf{x}},t)={\mathscr{J}}(0){\mathscr{G}}[{\bf{x}}-{\xi }_{0}\,\sin (\omega \tau )]+{\int }_{-\infty }^{t}d\tau \dot{{\mathscr{J}}}(t-\tau ){\mathscr{G}}[{\bf{x}}-{\xi }_{0}\,\sin (\omega \tau )]\mathrm{.}$$

The term ${\mathscr{G}}[{\bf{x}}-{\xi }_{0}\,\sin (\omega t)]$ can be re-written as:

6 $${\mathscr{G}}[{\bf{x}}-{\xi }_{0}\,\sin (\omega t)]={(2\pi )}^{-2}\int {d}^{2}q{\mathscr{G}}({\bf{q}}){e}^{-i{\bf{q}}\cdot [{\bf{x}}-{\xi }_{0}\sin (\omega t)]},$$

where ${\mathscr{G}}({\bf{q}})$ is the Fourier transform of the function ${\mathscr{G}}({\bf{X}})$. Now taking the time Fourier transform of ${\mathscr{G}}[{\bf{x}}-{\xi }_{0}\,\sin (\omega t)]$ we get:

7 $$\int dt{\mathscr{G}}\{{\bf{x}}-{\xi }_{0}\,\sin [\omega t]\}{e}^{-i{\rm{\Omega }}t}=\frac{1}{{(2\pi )}^{2}}\int {d}^{2}q{\mathscr{G}}({\bf{q}}){e}^{-i{\bf{q}}\cdot {\bf{x}}}\int dt{e}^{i[{\bf{q}}\cdot {\xi }_{0}\sin (\omega t)-{\rm{\Omega }}t]}\mathrm{.}$$

We need to calculate the integral $\int dt{e}^{i[{\bf{q}}\cdot {\xi }_{0}\sin (\omega t)-{\rm{\Omega }}t]}=\int dt{e}^{i{\bf{q}}\cdot {\xi }_{0}\sin (\omega t)}{e}^{-i{\rm{\Omega }}t}$. Using *θ* = *ωt*, it can be rewritten as

8 $$\int dt{e}^{i{\bf{q}}\cdot {\xi }_{0}\sin (\omega t)}{e}^{-i{\rm{\Omega }}t}=\frac{1}{\omega }\int d\theta {e}^{ir\sin \theta }{e}^{-i\alpha \theta }$$

where we have used $\alpha ={\rm{\Omega }}/\omega$ e $r={\bf{q}}\cdot {\xi }_{0}$. We can also write $\int d\theta {e}^{ir{\rm{\sin }}\theta }{e}^{-i\alpha \theta }={\int }_{-\pi }^{\pi }d\phi {e}^{ir{\rm{\sin }}\phi }{e}^{-i\alpha \phi }{\sum }_{k=-\infty }^{+\infty }{e}^{-2\pi ik\alpha }$, and noting that ${\sum }_{h=-\infty }^{+\infty }\delta (\alpha -h)={\sum }_{k=-\infty }^{+\infty }{e}^{-2\pi ik\alpha }$, we get

9 $$\int d\theta {e}^{ir\sin \theta }{e}^{-i\alpha \theta }=\sum _{k=-\infty }^{+\infty }\delta (\alpha -k){\int }_{-\pi }^{\pi }d\phi {e}^{ir\sin \phi }{e}^{-ik\phi }=2\pi \sum _{k=-\infty }^{+\infty }\delta (\alpha -k){J}_{k}(r)$$

where we have used that ${\int }_{-\pi }^{\pi }d\phi {e}^{ir\sin \phi }{e}^{-ik\phi }=2\pi {J}_{k}(r)$. Then, this leads to

10 $$\int dt{e}^{i{\bf{q}}\cdot {\xi }_{0}{\rm{s}}{\rm{i}}{\rm{n}}(\omega t)}{e}^{-i{\rm{\Omega }}t}=2\pi \sum _{k=-\infty }^{+\infty }\delta ({\rm{\Omega }}-k\omega ){J}_{k}({\bf{q}}\cdot {\xi }_{0})$$

where we have used that $\delta ({\rm{\Omega }}/\omega -k)=\omega \delta ({\rm{\Omega }}-k\omega )$. Replacing Eq. (10) into Eq. (7), we obtain

11 $$\int dt{\mathscr{G}}\{{\bf{x}}-{\xi }_{0}\,\sin [\omega t]\}{e}^{-i{\rm{\Omega }}t}=\sum _{k=-\infty }^{+\infty }2\pi \delta ({\rm{\Omega }}-k\omega ){A}_{k}({\bf{x}})$$

and, taking the inverse Fourier transform, we can rewrite Eq. (6) as:

12 $${\mathscr{G}}[{\bf{x}}-{\xi }_{0}\,\sin (\omega t)]=\sum _{k=-\infty }^{+\infty }{A}_{k}({\bf{x}}){e}^{ik\omega t}$$

where ${A}_{k}({\bf{x}})={\mathrm{(2}\pi )}^{-1}{\int }_{-1}^{1}ds{\mathscr{G}}({\bf{x}}-s{\xi }_{0}){B}_{k}(s)$ and ${B}_{k}(s)={(-i)}^{k}{T}_{k}(s){B}_{0}(s)$, with *T*_*k*_(*s*) being the Chebyshev polynomial of the first kind and *B*_0_(*s*) being ${B}_{0}(s)=2{(1-{s}^{2})}^{-\mathrm{1/2}}$, for $|s|\le 1$, and 0 otherwise. Substituting the relation (12) in Eq. (5), we finally obtain:

13 $$G({\bf{x}},t)=\sum _{k=-\infty }^{+\infty }\frac{{A}_{k}({\bf{x}})}{E(k\omega )}{e}^{ik\omega t}\mathrm{.}$$

In order to efficiently estimate *G*(**x**, *t*), let us focus preliminarily on the term *A*_*k*_(**x**). Indeed, it is possible to apply the Chebishev-Gauss quadrature rule to the integral term ${\int }_{-1}^{1}ds{\mathscr{G}}({\bf{x}}-s{\xi }_{0}){T}_{k}(s){(1-{s}^{2})}^{-\mathrm{1/2}}$ at *n* nodes so that *A*_*k*_(**x**) can be numerically estimated as:

14 $${A}_{k}({\bf{x}})=\frac{1}{n}\sum _{j\mathrm{=1}}^{n}\,\cos (k(\frac{2j-1}{2n}\pi )){\mathscr{G}}({\bf{x}}-{\xi }_{0}{\rm{c}}{\rm{o}}{\rm{s}}(\frac{2j-1}{2n}\pi ))\mathrm{.}$$

Consequently, Eq. (13) can be rewritten as:

15 $$G({\bf{x}},t)=\sum _{k=-\infty }^{+\infty }{U}_{k}{e}^{ik\omega t}$$

where *U*_*k*_ is equal to ${U}_{k}={[nE(k\omega )]}^{-1}{\sum }_{j\mathrm{=1}}^{n}\,\cos [k(2j-1)\pi /(2n)]{\mathscr{G}}\{{\bf{x}}-{\xi }_{0}\,\cos [(2j-1)\pi /(2n)]\}$. Now, we could directly calculate *G*(**x**, *t*) by means of a numerical estimation of the series in Eq. (15). However, in this paper, we propose a much more efficient approach. Indeed, what Eq. (15) says is that *G*(**x**, *t*) can be expressed as a Fourier series whose coefficient are *U*_*k*_. Consequently, if we generate an array $U=[{U}_{k=-N}\mathrm{,\; ...,}{U}_{k\mathrm{=0}}\mathrm{,\; ...,}{U}_{k=N}]$, we can obtain the array $G=[G({\bf{x}},{t}_{k=-N})\mathrm{,\; ...,}\,G({\bf{x}},{t}_{k\mathrm{=0}})\mathrm{,\; ...,}\,G({\bf{x}},{t}_{k=N})]$, i.e. the Green’s function for 2*N* + 1 values of the time *t*, simply by using the Inverse Fast Fourier Transform. In comparison with the estimation in the time domain of the series in Eq. (15), such an approach makes the search for the converged value of *G*(**x**, *t*) easier and speeds the entire calculation of orders of magnitude also when the frequency *ω* is small and computing in the time domain tends to be very slow. Incidentally, it should be observed that the final expression for *G*(**x**, *t*) relies on the factorization, performed in Eq. (1), of the integral equation kernel in two terms, that are ${\mathscr{G}}({\bf{x}})$ and ${\mathscr{J}}(t)$. This is allowed due to the assumption on the rheology of the contacting solids, which are considered homogeneous linear viscoelastic. In the case of materials exhibiting a more general time-dependent behaviour, given the periodicity of the system, *G*(**x**, *t*) could be again expressed as a Fourier series, whose coefficients would depend on the particular conditions marking the bodies in contact. This is, however, out of the scope of the paper.

As suggested for the elastic case in refs 24 and 25. and for viscoelastic systems in ref. 12, once the Green’s function *G*(**x**, *t*) is known, we can mesh the contact domain in *M* square cells and, then, discretize Eq. (3) by assuming that in each boundary element the normal stress *σ* is constant and equal to *σ*_*j*_. The displacement is, then, measured at the centre **x**_*i*_ of the each *i*-th square and is equal to ${u}_{i}=u({{\bf{x}}}_{i},t)$. Ultimately, Eq. (3) is reduced to the following linear system:

16 $${u}_{i}(t)={L}_{ij}(t){\sigma }_{j}$$

where *L*_*ij*_(*t*) is equal to $G({{\bf{x}}}_{i}-{{\bf{x}}}_{j},t)$. Indeed, the contact problem can be solved by using the iterative technique developed in ref. 24. for elastic contacts, thus providing contact areas, stresses and strains. Interestingly, the method does not require any discretization of the time domain as *t* is treated as a parameter. Furthermore, once the solution is known in terms of stresses and strains, following the approach commonly used in viscoelastic contact mechanics, stated in ref. 12, it is straightforward to calculate the viscoelastic interfacial tangential force as:

17 $${F}_{T}={\int }_{D}{d}^{2}x\sigma ({\bf{x}})\frac{\partial u}{\partial x}$$

Finally, we observe that *G*(**x**, *t*) has been calculated under the assumption that the shape of the stress field at the interface, which is generally equal to $\sigma ({\bf{x}},t)=\sigma [{\bf{x}}-{\xi }_{0}\,\sin (\omega t),t]$, does not change during the reciprocating motion, *i.e*. $\sigma ({\bf{x}},t)=\sigma [{\bf{x}}-{\xi }_{0}\,\sin (\omega t)]$. As widely discussed in ref. 21, this is rigorously equivalent to require that $|\partial \sigma /\partial t|/(|{\xi }_{0}\cdot \nabla \sigma |\omega )\ll 1$ and, consequently, as simple dimensional estimations reveal^21^, it is equivalent to assume that ${a}_{0}/|{\xi }_{0}|\ll 1$, where *a*_0_ is the characteristic dimension of the contact region.

## Results and Discussion

Let us start focusing briefly on the properties of the Green’s function *G*(**x**, *t*), whose formulation has been shown to be parametrically dependant on the time. Indeed, given the scope of this paper, i.e. damping in viscoelastic reciprocating contacts, it is important to analyze the displacement field since the amount of dissipated energy is proportional to the region within the material, which undergoes time-dependent deformations^12, 20^. The Green’s function, being just the displacement produced by a concentrated force, represents, then, the simplest displacement distribution that can be obtained.

**Figure 1:**
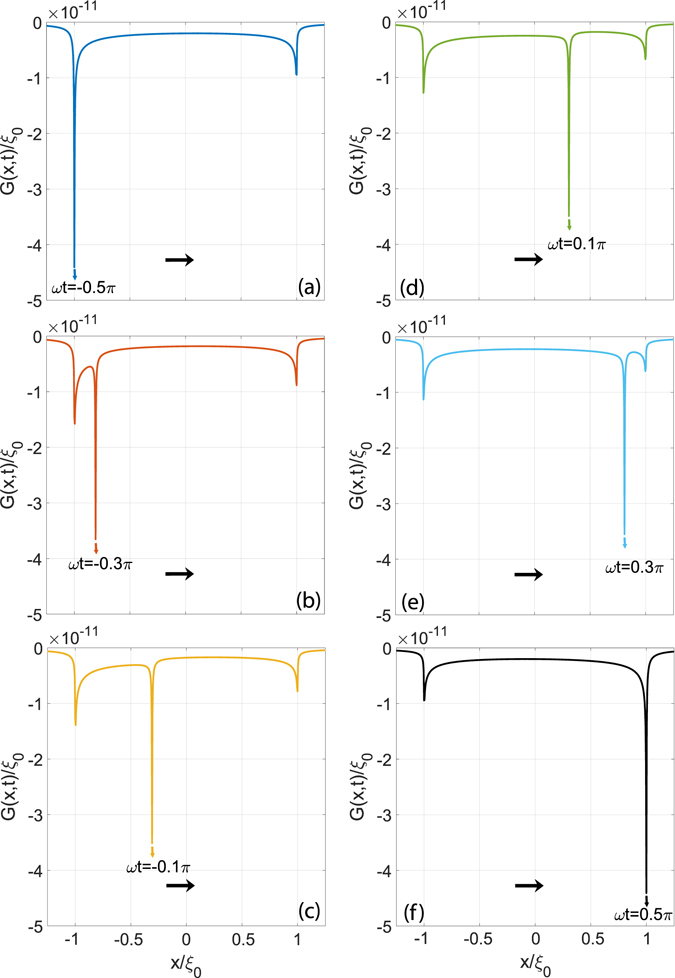
The quantity $G(x,y=\mathrm{0,}\,t)/R$ as a function of the dimensionless abscissa $x/{\xi }_{0}$ for several values of the dimensionless time $\omega t\in [-\pi \mathrm{/2,}\,\pi \mathrm{/2}]$.

Now, to carry out the analysis, we employ a simple one relaxation time material with the high frequency modulus *E*_∞_ equal to ${E}_{\infty }={10}^{8}{\rm{Pa}}$, the ratio ${E}_{\infty }/{E}_{0}=11$ and the Poisson ratio being *v* = 0.5. Furthermore, calculations are executed for an amplitude ${\xi }_{0}=0.01$ m and a dimensionless angular frequency of the reciprocating motion equal to *ωτ* = 5, being *τ* the relaxation time of the viscoelastic material. Figure 1 shows the evolution of the Green’s function for different values of the time $\omega t\in [-\pi \mathrm{/2,}\,\pi \mathrm{/2}]$. An arrow refers, in each case, to the current position of the force. At $\omega t=-\pi \mathrm{/2}$ the force has just reached the left dead-point and starts moving from left to right: the speed grows and, consequently, the material stiffens and the displacement decreases. Later on, for time larger than $\omega t=0$, the speed starts to decrease until the force stops for $\omega t=\pi \mathrm{/2}$. We observe that the displacement distribution shows three peaks: one corresponds to the current position of the force; the other two are found at the dead-points and are due to a typically viscoelastic delay in the deformation recover. Ultimately, the material is not able to fully relax and, consequently, to recover the viscoelastic deformation during a time interval comparable to the oscillation period.

**Figure 2:**
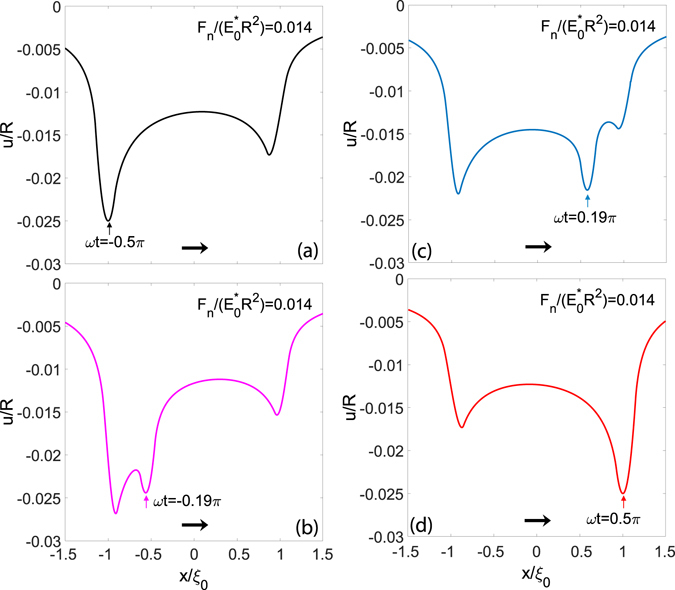
The dimensionless normal displacements $u(x,y=\mathrm{0)/}R$ as a function of the dimensionless abscissa $x/{\xi }_{0}$ in the case of the reciprocating motion of a sphere over a viscoelastic half-space. Calculations are carried out for a given normal force ${F}_{n}/{R}^{2}{E}_{0}^{\ast }=0.014$, an amplitude ${\xi }_{0}/R=1$ and several values of the dimensionless time $\omega t\in [-\pi \mathrm{/2,}\,\pi \mathrm{/2}]$.

The occurrence of multi-peaked displacement distributions is intrinsically related to viscoelasticity and characterizes also more complicated loading conditions, as for example when a rigid punch moves over regions that are still relaxing. To show this, let us study the contact of a rigid sphere of radius *R* which is in reciprocating sliding over a viscoelastic material with the following motion law ${\bf{x}}(t)=[{\xi }_{0}\,\sin (\omega t)\mathrm{,\; 0}]$. Given a specific dimensionless applied normal load ${F}_{n}/{R}^{2}{E}_{0}^{\ast }=0.014$ and a stroke ${\xi }_{0}/R=1$, Fig. 2 shows the evolution of the dimensionless displacements, *u*(*x*)/*R*, at the centre of the contact as a function of the abscissa $x/{\xi }_{0}$ and for different values of $\omega t\in [-\pi \mathrm{/2,}\,\pi \mathrm{/2}]$.

**Figure 3:**
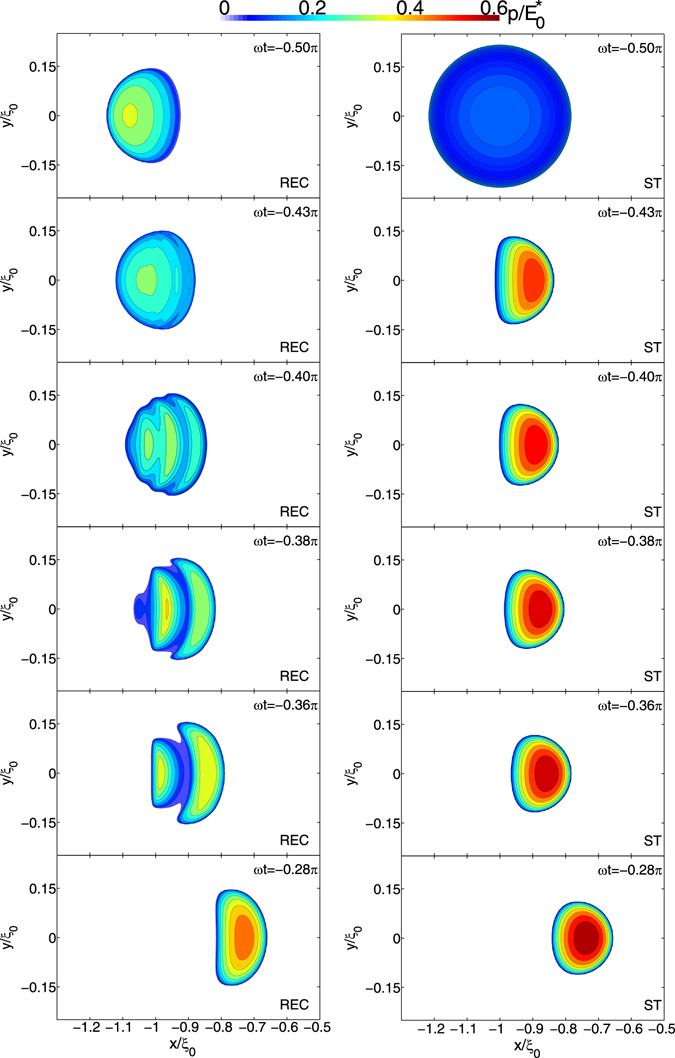
The contact area and the contour plots of the normalized contact pressure distributions, $p/{E}_{0}^{\ast }$, for several values of *ωt*. Calculations are carried out with the viscoelastic reciprocating theory (*on the left*) and the steady-state approach (*on the right*).

Coherently with what we observed before when looking at the Green’s function, upon reversal of the sliding direction, the punch moves over a viscoelastic region that has not yet had the time to recover the deformation after the previous contact of the rigid body. Furthermore, as the speed increases, the material stiffening is not negligible anymore and a marked decrease of the penetration is noticed. Ultimately, three different deformation peaks are observed in the path covered between the two dead-points: one corresponds to the current position of the punch, and the other two, being close to the dead-points, occur because the material does not fully recover the deformation in the oscillation period. Interestingly,we notice that such an interplay between the displacement fields provoke stress distributions, which show, upon the motion inversion, the jagged trend observed in Fig. 3. Indeed, starting from *ωt* = −*π*/2, although the sliding speed goes to zero, the interfacial normal stress distribution has an asymmetric shape, marked by a peak on the left of the contact patch: this is due to a tipically viscoelastic time-delay which prevents the material to relax immediately when the sliding speed vanishes. Clearly, such an effect cannot be embedded with a steady-state approch, which, for a speed equal to zero, predicts the static Hertzian solution. Now, as the sphere starts moving to the right, the peak shown by the reciprocating model at *ωt* = −*π*/2 does not disappear immediately but has to gradually decrease. At the same time, the punch is moving towards the right and, as shown also in steady-state conditions, a peak at the leading edge has to appear in the pressure distribution. Furthermore, at the center of such distribution, in correspondence of the maximum of the displacement field in the contact area, the pressure must still preserve an Hertzian-like trend. As a result, we have the multi-peaked pressure distributions observed in Fig. 3: the deviation from the steady-state predictions is evident and, interestingly, is significant also at *ωt* = −0.28*π*, i.e. when the punch is far enough from the inversion point and the pressure has evolved towards a smoother distribution. Indeed, this a further evidence of the necessity of considering the viscoelastic reciprocating properties.

Now, in order to relate such a mechanical behavior with viscoelastic tangential forces and with the energy dissipation, let us identify the parameters that govern the phenomenon. Naturally, the ratio ${E}_{\infty }/{E}_{0}$ plays an important role since it quantifies the degree of viscoelasticity: if ${E}_{\infty }/{E}_{0}=1$, it is straightforward to show that the material is perfectly elastic. Indeed, due to the sum rule^12^, we can write: $\frac{1}{{E}_{0}}-\frac{1}{{E}_{\infty }}=\frac{2}{\pi }{\mathscr{P}}{\int }_{0}^{+\infty }d\omega \frac{1}{\omega }Im\frac{1}{E(\omega )}$. However, more specifically related to the reciprocating motion and, therefore, to parameters like the stroke ${\xi }_{0}$, the frequency *ω* and the contact area *a*_0_, we find two different dimensionless groups^21^, which govern the problem. The first one is Π = *τ*/*t*_0_, where ${t}_{0}={a}_{0}/\omega {\xi }_{0}$ and *a*_0_ is the Hertzian contact radius, and compares the relaxation time *τ* with the time *t*_0_ needed by the sphere to cover a distance *a*_0_. Π can be seen, indeed, as the maximum dimensionless speed reached by the punch during its motion since it is equal to ${\rm{\Pi }}=\omega {\xi }_{0}\tau /{a}_{0}={v}_{{\rm{\max }}}\tau /{a}_{0}$. When Π ≪ 1, we are then in soft elastic conditions; for very large values of Π, then, we are in the glassy elastic regime in the most part of the track, but close to the inversion points, where the speed vanishes, some viscoelastic effects may be present. The second dimensionless parameter, governing the phenomenon, is the dimensionless frequency ${\rm{\Xi }}=\omega \tau =2\pi \tau /T$. Given a regime where viscoelasticity plays an important role, i.e. Π ≈ 1, if Ξ < 1, the reciprocating motion occurs on time-scales longer than the material relaxation time *τ* and the system behaves like in the steady-state case^12^. If Ξ ≈ 1, the system will show the significant effects specific of the reciprocating motions, as previously enlightened in Fig. 2. Finally, for very large values of Ξ ≫ 1, the punch carries out very fast reciprocating oscillations with an almost negligible stroke and again the high frequency elastic behavior will be recovered. Incidentally, we observe that, because of the constant shape assumption, which requires ${\rm{\Xi }}/{\rm{\Pi }}={a}_{0}/{\xi }_{0}\ll 1$, our methodology should not be utilized to predict the behavior of the system when ${\rm{\Xi }}/{\rm{\Pi }}={a}_{0}/{\xi }_{0}\gg 1$. However, when Ξ/Π ≫ 1 the behavior of the system is elastic, i.e. memory effects disappears, and the condition ${\rm{\Xi }}/{\rm{\Pi }}={a}_{0}/{\xi }_{0}\ll 1$ becomes needless, as the purely elastic regime is intrinsically embedded in the proposed methodology. In fact, the Green’s function *G*(**x**, *t*) in Eq. (3), for large Ξ/Π asymptotically converges to a time-indipendent purely elastic Green’s function *G*(**x**) provided by the Boussinesq solution^26^.

**Figure 4:**
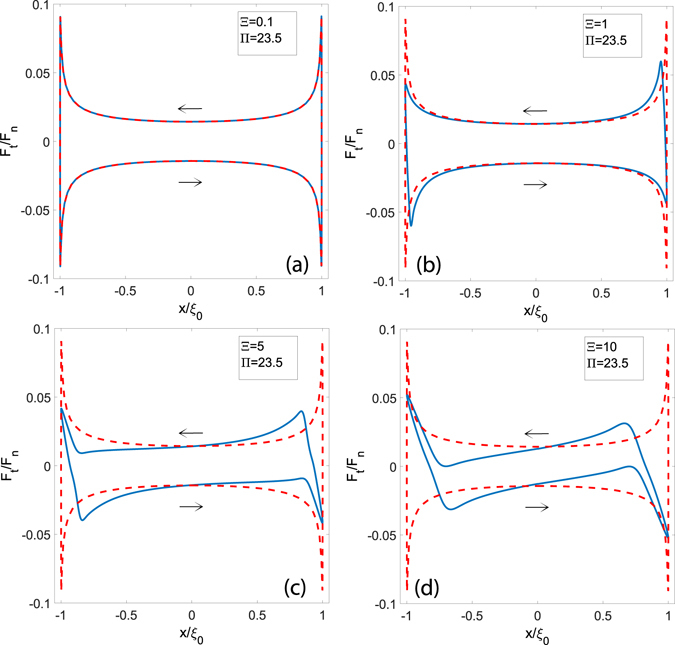
The dimensionless tangential force *F*_*t*_/*F*_*n*_ as a function of the dimensionless abscissa for several values of Ξ and Π = 23.5. Blue lines refer to results obtained by means of the viscoelastic reciprocating approach, whereas red dotted lines are obtained employing a classic steady-state model.

Let us, now, study the influence of the parameters Ξ and Π on the tangential forces during the cycle. In Fig. 4, after fixing the maximum speed Π equal to Π = 23.5, the dimensionless tangential force *F*_*t*_/*F*_*n*_ is plotted as a function of the dimensionless abscissa $x/{\xi }_{0}$, which identifies the position of the sphere center along the path, for different values of the frequency Ξ. In particular, blue lines refer to the simulations obtained by means of the reciprocating model, whereas values on the dotted red lines are computed by employing a steady-state model^12^ given, in each instant *t* of the cycle, a rolling velocity $v(t)=|{\xi }_{0}|\omega \,\cos (\omega t)$. Note that the steady-state model neglects any reciprocating effect as the parameter Ξ does not play any role in this case.

As expected, for low values of Ξ, the reciprocating solution approaches the steady-state regime. A different behavior occurs, however, when Ξ is increased: upon the motion inversion, due to the interaction of the punch with a still deformed area, the actual reciprocating behavior strongly deviates from what is predicted by the steady-state model. In detail, because of the reciprocating interplay, when the motion is inverted, the tangential force *F*_*t*_/*F*_*n*_ has the same direction as the sliding speed and the entire dissipation cycle appears twisted. Furthermore, as Ξ and Ξ/Π grow, the cycle area tends to reduce in agreement with what expected theoretically, i.e. that the system approaches an elastic behaviour.

Now, after recalling that, in each point of the cycle, the punch has a speed *v*(*t*), let us introduce the dimensionless dissipated work per cycle *δ*:

18 $$\delta =\frac{\oint {F}_{t}vdt}{{F}_{n}{\xi }_{0}}$$

**Figure 5:**
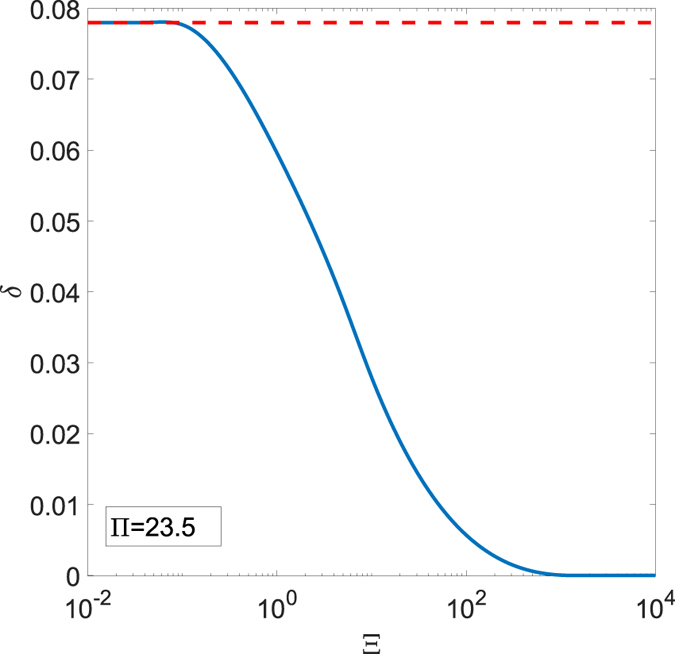
The dimensionless dissipated work *δ* as a function of the frequency Ξ for a fixed value of Π = 23.5. The blue lines refers to results obtained by means of the viscoelastic reciprocating approach, whereas the red dotted line is obtained employing a classic steady-state model.

In Fig. 5, fixed Π, *δ* decreases with Ξ and goes to zero for Ξ ≫ 1. Therefore, employing an approach based on steady-state assumptions, as the one followed usually when designing dampers (see e.g. ref. 27.), may lead to a massive overestimation of the dissipated power and, then, to a poor design.

**Figure 6:**
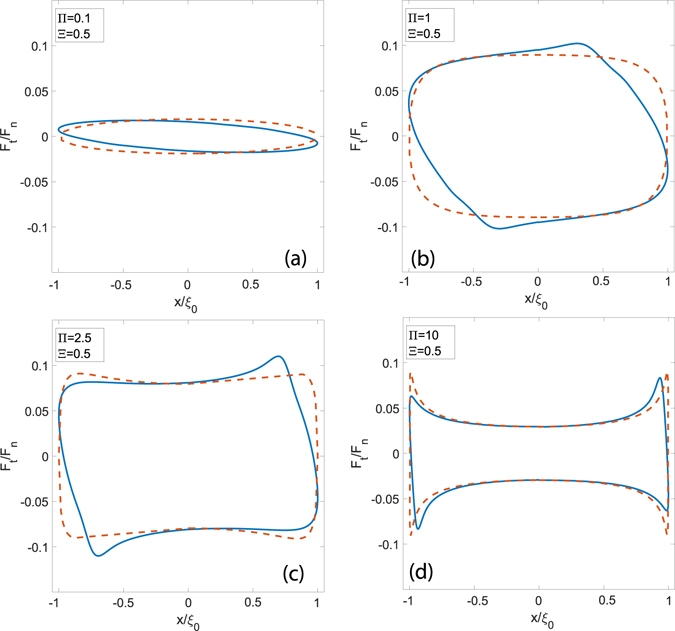
The dimensionless tangential force *F*_*t*_/*F*_*n*_ as a function of the dimensionless abscissa for several values of Π and Ξ = 0.5. Blue lines refer to results obtained by means of the viscoelastic reciprocating approach, whereas red dotted lines are obtained employing a classic steady-state model.

At this point, in order to study the influence of the other parameter, i.e. the maximum dimensionless speed in the cycle Π, it may be interesting to fix the frequency Ξ and sweep a range of values for Π. Let us preliminarly recall that, in steady-state conditions, where friction force and friction coefficient are functions only of the dimensionless speed *vτ*/*a*_0_, simple dimensional considerations (see ref. 12), show that the maximum friction coefficient and dissipation are obtained for a dimensionless speed equal to $\zeta \approx {\pi }^{-1}\sqrt{({E}_{\infty }/{E}_{0})}$. Now, in a reciprocating cycle, depending on the ratio ${\rm{\Pi }}/\zeta$, we will find different shapes for the hysteretic curve. Indeed, as shown in Fig. 6, if ${\rm{\Pi }} < \zeta$, the viscoelastic tangential force *F*_*t*_/*F*_*n*_ continuously increases when the speed grows in the cycle. The latter is, then, convex. In fact, since the instantaneously velocity *v*(*t*) is always smaller than the $\zeta {a}_{0}/\tau$, the viscoelastic material is excited in a frequency range where the loss moduls of the material (i.e. $Im[E(\omega )]$) always grows with the frequency^22, 23^: this makes, indeed, friction an increasing function of the rolling velocity. On the contrary, when ${\rm{\Pi }} > \zeta$, *F*_*t*_/*F*_*n*_ reaches again its maximum for $v(t)=\zeta {a}_{0}/\tau$, but there will exist a time range where *v*(*t*) exceeds $\zeta {a}_{0}/\tau$, thus leading the viscoelastic material to be exicited on the decreasing branch of the loss-modulus vs. frequency curve. As a matter of fact, the hysteretic curve tends to look spiked close to the dead-points. Such a trend is found both when employing the reciprocating method and in steady-state conditions, but significant quantitave differences occur between the two approaches. As previously discussed, this is due to the interplay between not fully relaxed regions of the viscoelastic half-space.

**Figure 7:**
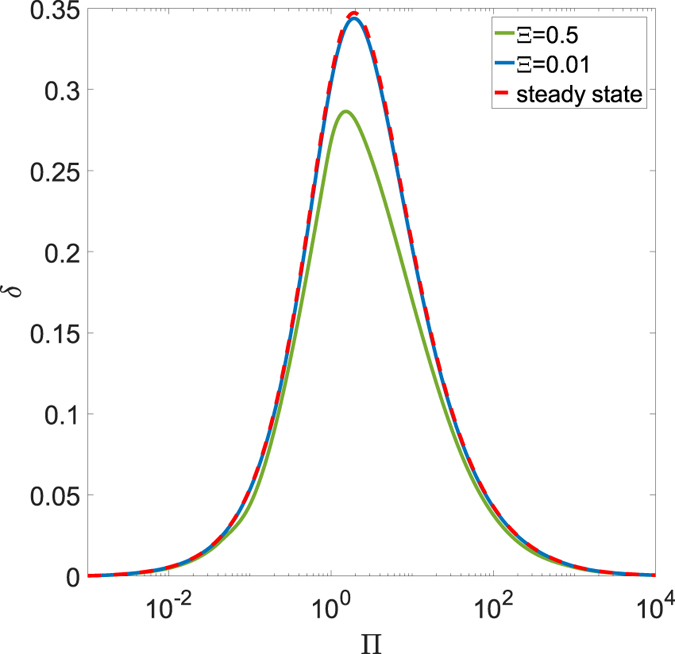
The dimensionless dissipated work *δ* as a function of the speed Π and for several values of the frequency Ξ.

**Figure 8:**
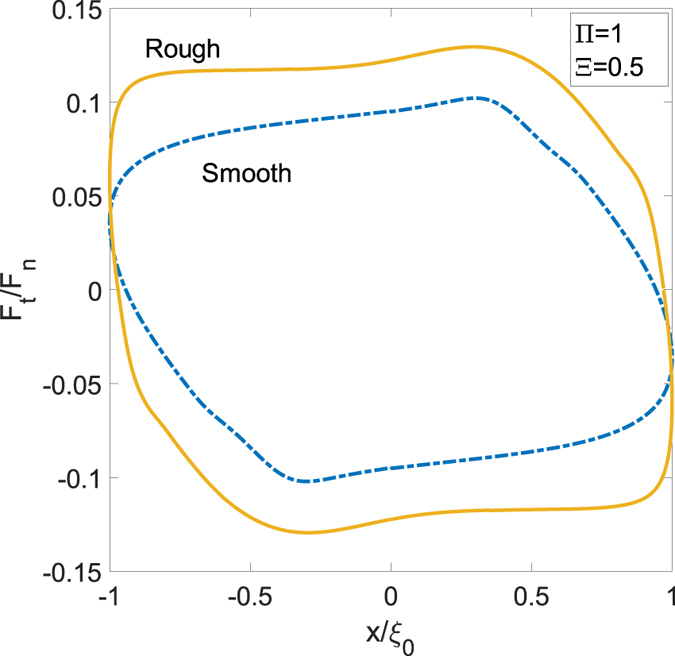
The dimensionless tangential force *F*_t_/*F*_n_ as a function of the dimensionless abscissa for a smooth and a rough sphere.

When looking at the dissipated work *δ*, from a quantitative point of view, we notice that *δ* decreases with Π and goes to zero once the system approaches the soft elastic regime. On the other side, for very large values of Π, the system is in the glassy elastic regime during the most part of the cycle, apart from very narrow regions close to each dead-point, where the punch arrests and inverts its motion. In Fig. 7, we can focus on *δ* and observe a bell-shaped curve: as expected, the real dissipation computed by the viscoelastic reciprocating model is smaller in comparison with the predictions carried out by means of the steady-state approach. Such a scenario confirms the necessity of the methodology introduced in this paper to account for the actual interactions in the reciprocating dynamics. This is even more true when the real rough spectra of the contacting surfaces is accounted for. The presence of a rough interface covering many orders of magnitude has, undoubtedly, a strong importance: as already shown in the steady-state case^17^, the viscoelastic dissipation grows by increasing the number of rough scales. In detail, given a self-affine fractal surface numerically generated, for example, by means of the spectral method described in ref. 25, viscoelastic friction grows significantly with the short-length cut-off wavevector *q*_1_ = *2πN/L*_0_, where *L*_0_ is the roll-off length and *N* the number of scales. Such a trend is confirmed for the reciprocating conditions in Fig. 8, where the tangential force *F*_*t*_/*F*_*n*_ is shown both for a smooth and for a rough sphere. This rough interface has been obtained spreading on the smooth sphere a fractal self-affine surface whose spectral components are in the range *q*_0_ < *q* < *q*_1_, where *q*_0_ = *2π/R* and *q*_1_ = *Nq*_0_. In particular, results in Fig. 8 are obtained with *N* = 32 and show, in addition to the increased dissipation, a different shape due to the presence of the roughness.

## Conclusions

In this paper, we present a numerical methodology capable of providing accurate predictions in the case of reciprocating motion between viscoelastic solids. Implications for the damping of rubber-like components are enormous since reliable numerical predictions are finally possible. In particular, in the simple case of the reciprocating contact of a sphere over a viscoelastic half-space, the phenomenon is shown dependent just on two parameters, that are the maximum dimensionless speed Π reached during the motion and the dimensionless frequency Ξ. To this extent, it is crucial to notice that, if the analysis of the viscoelastic dissipation was conducted as usual done in literature with a steady-state approach (see e.g. ref. 27.), such a dependence on Ξ would be completely neglected. This issue is particularly critical for Ξ/Π ≫ 1, when the viscoelastic damping has to go to zero, as correctly predicted by the reciprocating model.

The damping overestimation, intrinsically related to the steady-state approach, is, indeed, related to the poor description that such stationary models produce on the reciprocating physics. Indeed, upon the motion inversion, the punch gets into contact with a region of viscoelastic half-space, which is still deformed and has to recover the deformation yet. This produces the multi-peaked displacement distribution shown in the paper and, more interestingly, gives origin to the strong dependence on the frequency Ξ. All this is ignored from the steady-state point of view, which ultimately ignores all the reciprocating loading history of the material. This is even more significant when considering the surface roughness, which, as in any phenomenon related to viscoelastic contact mechanics, plays a crucial role. The viscoelastic material is stressed over a frequency range, whose width is strictly related to the rough spectra^17^

, and, consequently, the interplay between regions not fully relaxed cannot be neglected.
